# Redox Proteomics Identification of Oxidatively Modified Myocardial Proteins in Human Heart Failure: Implications for Protein Function

**DOI:** 10.1371/journal.pone.0035841

**Published:** 2012-05-14

**Authors:** Maura Brioschi, Gianluca Polvani, Pasquale Fratto, Alessandro Parolari, Piergiuseppe Agostoni, Elena Tremoli, Cristina Banfi

**Affiliations:** 1 Centro Cardiologico Monzino IRCCS, Milan, Italy; 2 Department of Pharmacological Sciences, University of Milan, Milan, Italy; 3 Department of Cardiovascular Science, University of Milan, Milan, Italy; 4 Niguarda Hospital, Milan, Italy; 5 Department of Clinical Care and Respiratory Medicine, University of Washington, Seattle, Washington, United States of America; National Taiwan University Hospital, Taiwan

## Abstract

Increased oxidative stress in a failing heart may contribute to the pathogenesis of heart failure (HF). The aim of this study was to identify the oxidised proteins in the myocardium of HF patients and analyse the consequences of oxidation on protein function. The carbonylated proteins in left ventricular tissue from failing (n = 14) and non-failing human hearts (n = 13) were measured by immunoassay and identified by proteomics. HL-1 cardiomyocytes were incubated in the presence of stimuli relevant for HF in order to assess the generation of reactive oxygen species (ROS), the induction of protein carbonylation, and its consequences on protein function. The levels of carbonylated proteins were significantly higher in the HF patients than in the controls (p<0.01). We identified two proteins that mainly underwent carbonylation: M-type creatine kinase (M-CK), whose activity is impaired, and, to a lesser extent, α-cardiac actin. Exposure of cardiomyocytes to angiotensin II and norepinephrine led to ROS generation and M-CK carbonylation with loss of its enzymatic activity. Our findings indicate that protein carbonylation is increased in the myocardium during HF and that these oxidative changes may help to explain the decreased CK activity and consequent defects in energy metabolism observed in HF.

## Introduction

There is experimental evidence showing increased oxidative stress in a failing heart and that this contributes to the pathogenesis of the myocardial remodelling that leads to heart failure (HF) [1]. Patients with chronic HF have high levels of oxidative stress markers, which have been related to myocardial dysfunction and the overall severity of HF [1]. Increased levels of lipid peroxidation markers and broached antioxidant reserves have both been reported in humans with HF [2], [3]: in particular, the pericardial levels of 8-iso-prostaglandin F2alpha increase with the functional severity of HF and are associated with left ventricular (LV) dilation [2], and the cardiac tissue levels of 4-hydroxy-2-nonenal correlate with systolic dysfunction [4]. Furthermore, Dhalla *et al*. have reported that oxidative stress in the myocardium of guinea pigs increases during the transition from compensatory hypertrophy to HF subsequent to chronic pressure overload [5].

These findings are not surprising because it is known that oxidative damage can be caused by a number of factors associated with HF, including high plasma catecholamine levels, increased cardiac sympathetic tone, microvascular reperfusion injury, cytokine stimulation, and mitochondrial deoxyribonucleic acid mutations. Reactive oxygen species (ROS) are produced in the failing myocardium by different sources, and cause hypertrophy, injury, apoptosis, and necrosis in cardiomyocytes [6]–[12].

ROS can modify various biological molecules, such as carbohydrates, lipids, nucleic acid and proteins [13]. Proteins are the main targets because they are abundant in biological systems and primarily responsible for the processes of most cell functions; it has also been estimated that they can scavenge the majority (50–75%) of ROS [14]. Most protein modifications are irreparable, and oxidative changes in protein structure can have a wide range of downstream functional consequences as they may inhibit enzymatic or binding activities, increase susceptibility to aggregation and proteolysis, increase or decrease cell uptake, and alter immunogenicity [14].

The most widely studied oxidative stress-induced protein modification is the formation of carbonyl groups on amino acid residues [14]. The amount of carbonyls is considered a marker of oxidative stress, and is used to quantify the level of oxidative damage in polypeptide chains [14]. Moreover, analysing protein carbonyl groups may have some advantages over other methods: their formation is a common product of protein oxidative reactions, and they are produced early, remain relatively stable, and are induced by almost all types of ROS [15].

It is now known that protein oxidation plays an essential role in the pathogenesis of a large number of degenerative diseases [16]. However, a complete analysis of protein carbonylation in the myocardium of HF patients is still lacking.

Aim of this study is to gain a deeper insight in the effects of ROS on protein carbonylation in the human failing heart, and, in particular: a) to analyse the presence of carbonylated proteins in the myocardium of HF patients; b) to identify the targets of carbonylation using a proteomic approach; and c) to identify the molecules responsible for protein oxidation in *in vitro* cultured cardiomyocytes.

## Materials and Methods

Unless otherwise specified, all reagents and chemicals were purchased from Sigma-Aldrich**,** Milan, Italy.

### Ethic Statement

The study protocol was approved by the Ethics Committees of Monzino Cardiologic Center and Niguarda Hospital, and was conducted in accordance with the principles laid down in the Declaration of Helsinki.

### Subjects and Heart Tissue Samples

Non-failing left ventricular apical tissue was harvested from 13 donor hearts excluded from transplantation for technical reasons (mean ejection fraction: 65±4%) at the Homograft Bank of Monzino Cardiologic Center (Milan, Italy) and immediately frozen at −80°C. The mean age of the donors was 46±2.2 years; seven were male.

Non necrotic tissue from the apex of 14 failing hearts was obtained from patients undergoing cardiac transplantation at Niguarda Hospital, all of whom gave their written informed consent. The cause of heart failure was idiopathic dilated cardiomyopathy (n = 7), and ischemic cardiomyopathy (n = 7). The mean age of the patients was 46±2.2 years; six were male. All of the patients had an ejection fraction of <20%, but none of them was on the urgent heart transplant list. Moreover, only two had been treated with a long term left ventricular assist device or had received chronic intravenous inotropic support during the seven days preceding transplantation. Heart failure therapy was optimised in all cases and included angiotensin-converting enzyme inhibitors (77%), β-blockers (70%), diuretics (85%), antiarrhythmic drugs (15%), antiplatelets (62%), anticoagulant drugs (38%). The comorbidities were hypertension (46% of the patients) and diabetes (23%).

### Protein Extraction from Heart Tissue

The heart tissue specimens were ground to a powder under liquid nitrogen using a mortar and pestle, resuspended in 20 mmol/L HEPES, 20 mmol/L NaCl, 5 mmol/L EDTA, 1% w/v CHAPS with protease inhibitors (Sigma-Aldrich) (1 g of tissue in 7 mL), homogenised at setting 5 of a Polytron homogenizer (three 10 s bursts), sonicated using a Branson Sonifier 250 (three times for 20 s each), and centrifuged at 13000×*g* for 10 min at 4°C. The supernatants were treated with protein-L-agarose for 45 min (20 microlitres per 1 mL) in order to eliminate contaminating immunoglobulins. After centrifugation, the supernatants were recovered and protein concentration was measured using Bradford’s assay [17].

### Quantification of Protein Carbonyl Groups

Cardiac protein carbonyls were measured using a Zentech PC test ELISA (Biocell, Auckland, New Zealand). Briefly, heart tissue protein lysate prepared as described above was reacted with 2,4-dinitrophenylhydrazine (DNPH) in order to convert the protein carbonyl groups to the corresponding 2,4-dinitrophenylhydrazones (DNP), which can be detected by means of an anti-DNP antibody. Absorbances were read using a 450 nm filter. The standard curve of oxidised albumin and the samples were assayed in triplicate; the intra- and inter-assay coefficients of variation were respectively less than 2.1% and 3.2%.

### Sample Preparation and 2-dimensional Electrophoresis for the Detection of Protein Carbonyls

The cardiac proteins were diluted in a buffer yielding final concentrations of 8 mol/L urea, 2 mol/L thiourea, 4% w/v CHAPS, 2% v/v carrier ampholytes, pH 3–10, 20 mmol/L Tris, 55 mmol/L DTT, and bromophenol blue. Two-dimensional electrophoresis (2-DE) was carried out in accordance with the manufacturer’s protocol (Protean IEF cell, Biorad, Milan, Italy), with 70 mm IPG ready strips, pH 3–10 non-linear gradient (Biorad, Milan, Italy), as described in the Methods S1. All of the analyses were made in duplicate: one gel was stained with Coomassie Colloidal Blue G250 for total protein analysis, and the other was electroblotted to a polyvinylidene fluoride (PVDF) membrane for DNP immunostaining to evaluate the carbonylated proteins as previously described [18]. The procedure was performed in triplicate for each sample in order to evaluate gel reproducibility and improve the reliability of the estimates of qualitative and quantitative changes in protein expression.

All of the images were scanned using a GS-800 densitometer (Biorad, Milan, Italy) before being analysed by means of Progenesis SameSpot software (Nonlinear Dynamics, Newcastle upon Tyne, UK).

Progenesis SameSpot software v 2.1 was used for gel alignment, spot detection, spot quantification, and normalisation for total spot volume in each gel, and the data were statistically analysed using the incorporated statistical package. Oxidation index was then calculated as the ratio between spot immunointensity divided by intensity of Colloidal Blue protein staining. The cut-off level for a differentially expressed oxidized protein was defined as at least a 1.5 fold increase or decrease in protein oxidation index.

Statistically significant between-group differences for each protein were computed using the non-parametric Wilcoxon Mann-Whitney test, and a p value of <0.05 was considered statistically significant. Proteins with altered immunoreactivity were identified by mass spectrometry (MS) after excision of the matching spot on the superimposed gel stained with Coomassie Blue.

### Mass Spectrometry Analysis

The selected proteins were in-gel reduced, alkylated, digested with trypsin as previously described [19] and the samples were analysed by liquid chromatography-mass spectrometry (LC-MS/MS), with the spectra being recorded by a hybrid quadrupole orthogonal acceleration time-of-flight Q-Tof mass spectrometer with an electrospray ionization (ESI) source (Synapt-MS, Waters corporation, Manchester, UK), connected to a Nano-Acquity UPLC system. Further details are available in the Methods S1.

### Cell Culture Experiments

The HL-1 cardiomyocytes were a kind gift of Professor W.C. Claycomb, LSU Health Sciences Center, New Orleans, LA, USA [20] and were cultured in complete Claycomb Medium supplemented with 10% fetal calf serum (FCS) (JRH Biosciences, Lenexa, KS, USA) and 100 µmol/L norepinephrine (Sigma-Aldrich, Milan, Italy), following Professor Claycomb’s instructions. The cells were starved in a medium without serum and norepinephrine for at least 24 h before incubation with the various compounds (isoprotenerol, phenylephrine, norepinephrine, angiotensin II, endothelin-1, and TNFα) in the same medium, to avoid the interference of serum factors which mask the stimulatory effects of agonists (data not shown).

### Formation of Intracellular Reactive Oxygen Species (ROS)

HL-1 cells cultured on 96-well plates were loaded with 10 µmol/L 2′,7′-dichlorofluorescein diacetate (DCFH-DA) for 1 h at 37°C and, after incubation, were washed in phosphate buffered saline (PBS) and exposed to the various compounds or vehicle for the indicated times. ROS production was measured on the basis of the intensity of DCF emission at 535 nm (excitation 485 nm) in a Mithras LP940 fluorescence spectrometer (Berthold Technologies Italia, Milan, Italy). The curve that remained after subtracting the fluorescent signal of untreated cells from that of treated cells was used to evaluate the stimuli-induced increase in fluorescence; the results are expressed as the percentage increase in fluorescence in comparison with the untreated cells.

### Immunocytochemical Analysis of Protein Carbonyls

Protein carbonyls were detected using the protocol described by Smith *et al* [21]. The treated cells were immediately fixed for 24 h at 4°C using a solvent mixture (methanol/chloroform/acetic acid 60/30/10; v/v/v) to remove cellular lipids. After removing the solvent mixture, the cells were incubated for 16 h at 4°C with DNPH (300 mg DNPH per 100 mL 96% ethanol containing 1.5% pure sulfuric acid) in order to convert the protein carbonyl groups to the corresponding 2,4-dinitrophenylhydrazones, which can be detected using an anti-DNP antibody. The residual DNPH was removed by first extensively washing the cells with PBS containing 1% FCS (washing buffer) and then incubating them for 30 min at 4°C in washing buffer. The primary polyclonal anti-DNP antibody (Sigma-Aldrich, Milan, Italy) was diluted 1∶200 in washing buffer for 1 h at 4°C, and after exhaustive washing, the cells were treated with a secondary Alexa fluor 488-labeled anti-rabbit antibody (Invitrogen, Milan, Italy) diluted 1∶100 in washing buffer for 1 h at 4°C. After removing the secondary antibody and washing, the cells were microscopically examined.

### CK Immunoprecipitation, Derivatisation by DNPH, SDS-PAGE and Immunochemical Detection of Carbonylation

To analyse CK carbonylation *in vitro*, HL-1 cells were treated with angiotensin II, phenylephrine or vehicle for 24 h, then collected in ice-cold PBS and centrifuged at 700×*g* for 3 min. The cell pellets were suspended in lysis buffer (50 mmol/L Tris, 150 mmol/l NaCl, 1% v/v Triton X-100, 1% w/v deoxycholate and 0.1% w/v SDS, pH 8), supplemented with a cocktail of protease inhibitors (Sigma-Aldrich), and lysed at 4°C for 30 min. The cell lysates were sonicated three times for 10 s on ice and centrifuged at 15000×*g* for 15 min. After centrifugation, the clarified supernatants were immunoprecipitated for 1 h at room temperature with anti M-CK antibody (2 µg/mg protein, Santa Cruz Biotechnology, Santa Cruz, CA, USA) cross-linked to the uniformly sized super-paramagnetic nanoparticle Bio-Adembeads Protein G (Ademtech, Pessac, France) as indicated in the manufacturer’s protocol. To avoid the co-elution of immunoglobulin, the antibody was cross-linked to the beads (2 µg per 20 µL of beads) after treatment with 200 mmol/L triethanolamine and 20 mmol/L dimethyl pimelimidate dihydrochloride as the cross-linker.

The Bio-Adembeads protein G cross-linked to the anti-CK antibody were then incubated with the cell lysate in order to form a magnetically labelled immune complex that can be easily separated from the liquid by removing the supernatant with a magnet. The immunoprecipitates were washed three times with PBS before elution with 50 mmol/L glycine, 0.65% Tween, pH 2.7.

The proteins were derivatised with 10 mmol/L DNPH (in 2 N HCl) for 1 h at room temperature. The DNPH-derivatised samples were neutralised with 10 mmol/L sodium phosphate buffer, pH 7.45, containing 0.1% w/v SDS, 30% v/v glycerol and 5% v/v 2-β-mercaptoethanol, and the proteins were separated by SDS-PAGE and blotted to PVDF membranes. Immunodetection was performed using biotinylated anti-DNP antibody (Invitrogen, Milan, Italy) and conjugated avidin-HRP (Biorad, Milan, Italy) or anti M/B-CK antibody (Santa Cruz Biotechnology, Santa Cruz, CA, USA). As a negative control, Bio-Adembeads Protein G was incubated with cell lysate without antibodies.

### Detection of S-nitrosylated CK

The occurrence of S-nitrosylated CK was assessed by means of the biotin-switch assay [22] using the S-nitrosylation protein detection kit (Cayman Chemical, Ann Arbor, MI, USA). This method is aimed at converting nitrosylated cysteines into biotinylated cysteines. Briefly, free thiols of proteins were blocked with the thiol-specific agent methylmethanethiosulfonate. Then S-nitrosothiols were selectively reduced by ascorbate to form thiols, which were reacted with N-[6-(biotinamido)hexyl]-3′-(2′-pyridyldithio)-propionamide, a sulfhydryl-specific biotinylating reagent. After derivatization the biotinylated/S-nitrosylated CK was immunoprecipitated as described above. Immunodetection was performed using a biotin detection reagent and anti M/B-CK antibody.

### CK Activity

CK activity was measured in 10 µg of cell extract or cardiac tissue homogenate using the EnzyChrom™ Creatine Kinase Assay Kit (BioAssay Systems’ QuantiChrom, Gentaur, Milan, Italy), which is based on enzyme-coupled reactions in which creatine phosphate and ADP are converted to creatine and ATP by CK. The generated ATP is used to phosphorylate glucose by hexokinase in order to generate glucose-6-phosphate, which is then oxidised by NADP in the presence of glucose-6-phosphate dehydrogenase. The produced NADPH, measured at 340 nm, is proportionate to the CK activity in the sample, and the data are expressed as percentage of CK activity in treated cells with respect to control cells. Purified human M-CK was from Sigma-Aldrich.

### Statistical Analysis

The numerical data are given as mean values±SD. The non-parametric Wilcoxon Mann-Whitney test was used to compare the controls and patients, with a p value of <0.05 being considered statistically significant. Statistically significant between-group differences in the *in vitro* experiments were computed using analysis of variance (ANOVA) followed by Tukey’s *post hoc* test in order to allow both multiple group and individual group-to-group comparisons; a p value of <0.05 was considered statistically significant. These statistical analyses were made using SPSS software (v 13.0, SPSS, Chicago, IL, USA).

## Results

**Figure 1:**
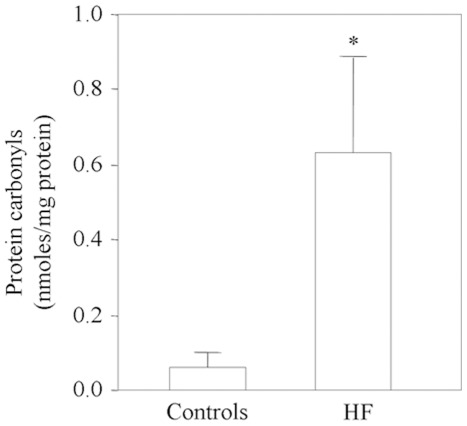
ELISA-measured protein carbonyl levels in the myocardium of 14 HF patients and 13 controls. *p<0.01 *vs* controls.

**Figure 2:**
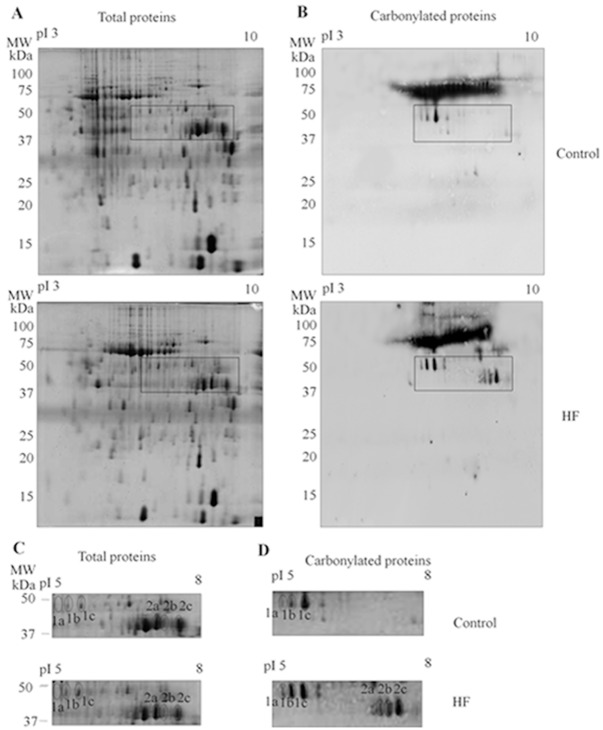
Representative 2-DE analysis of carbonylated cardiac proteins. (**A**): representative whole 2-DE gel stained with Coomassie Colloidal Blue to visualise all of the proteins from controls and patients with HF. (**B**): representative whole 2-DE gel of proteins from controls and patients with HF immunostained with the anti-DNP antibody. Expanded views of the “area of magnification” of the differentially carbonylated proteins from controls and HF patients stained with Coomassie Colloidal Blue (**C**) or immunostained with the anti-DNP antibody (**D**). The spot numbers indicated in the map are the same as those used for MS identification (**Table S1**).

Carbonylated protein levels measured by ELISA were significantly higher in the hearts of the HF patients than in the control hearts (p<0.01, Wilcoxon Mann-Whitney analysis) (**Fig. 1**), but the difference between the ischemic and idiopathic patients was not significant (0.60±0.32 nmoles/mg protein *vs* 0.87±0.24 nmoles/mg protein; p = 0.34). The carbonylated proteins were then identified by means of 2-DE combined with the high specificity of immunoblotting after the DNPH derivatisation of the carbonyl groups. Immunoblotting with anti-DNP antibody, performed on 14 left ventricular tissue samples from HF patients and 13 from healthy controls, showed that different isoforms of two proteins were carbonylated to a greater extent in the myocardium of the patients (**Fig. 2** and **Table S1**). The carbonylated protein spots were excised from the 2-DE gel, digested with trypsin, and analysed by means of LC-MS/MS, which identified them as α-cardiac actin (ACTC) and creatine kinase M-type (M-CK), which is only detectable in its oxidised form in failing hearts (**Table S1**). The analysis of CK activity revealed that it was significantly reduced in the myocardium of HF patients than in controls (88.9±10.4 U/mg protein *vs* 175±9.6 U/mg protein, p = 0.0001, respectively).

Considering that other forms of protein oxidation, such as those induced by reactive nitrogen species, could have an impact on CK activity we evaluated the occurrence of S-nitrosylation (i.e. the covalent attachment of nitric oxide to cysteine thiol) in the myocardium of controls and HF patients. S-nitrosylated proteins were selectively biotinylated, CK was immunoprecipitated and the S-nitrosylated form of CK was revealed with a biotin detection reagent. This analysis revealed that there are no difference in terms of S-nitrosylation of CK between controls and HF patients (**Fig. S1**). Furthermore, the total profile of S-nitrosylated proteins was very similar between control and HF patients (**Fig. S1**).

**Figure 3:**
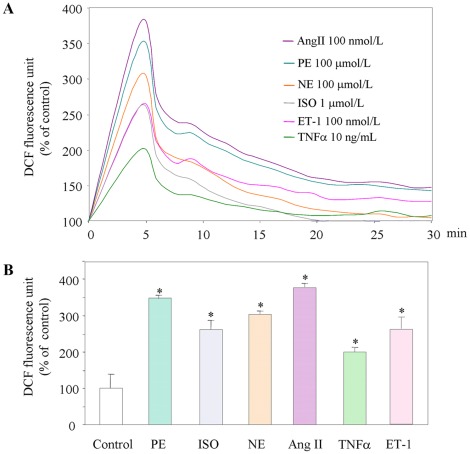
Intracellular generation of ROS in cardiomyocytes exposed to various compounds. (**A**) Relative DCF fluorescence of ROS generation. (**B**) Percentage increase in ROS generation vs control cells after 5 min. *p<0.01 vs control cells; n = 5. PE, phenylephrine; ISO, isoprotenerol; NE, norepinephrine; AngII, angiotensin II; ET-1, endothelin-1, TNFα, tumor necrosis factor α.

In order to identify the factors responsible for the increased formation of carbonylated proteins, *in vitro* cultured HL-1 cardiomyocytes were exposed to the β1 and β2 agonist isoprotenerol (0.1–1 µmol/L), the α1 agonist phenylephrine (10–100 µmol/L), the α1, α2 and β1 agonist norepinephrine (10–100 µmol/L), angiotensin II (10–100 nmol/L), endothelin-1 (10–100 nmol/L), and TNFα (1–10 ng/mL). Intracellular H_2_O_2_ formation measured on the basis of the intensity of DCF emission was monitored for 1 h. All of the tested compounds induced the rapid intracellular generation of H_2_O_2_, which subsequently gradually decreased to baseline levels (**Fig. 3**).

**Figure 4:**
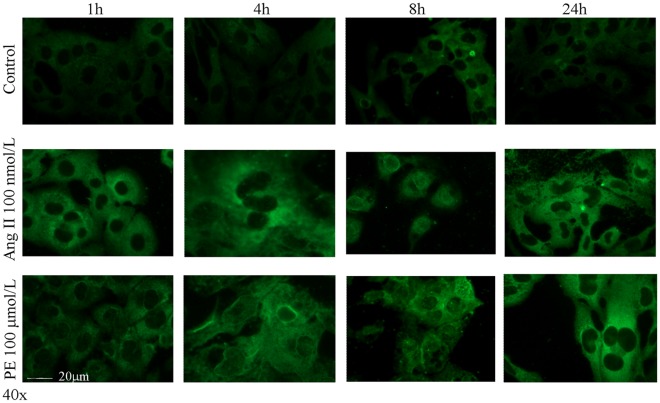
Immunofluorescence analysis of carbonylated proteins in cardiomyocytes after treatment with angiotensin II (AngII) or phenylephrine (PE). The cells were stained with anti-DNP antibody and visualised by means of a secondary antibody conjugated with Alexa Fluor dye 488. Representative of three independent experiments.

We then looked for the presence of protein carbonyls in HL-1 cells exposed for different times to angiotensin II or phenylephrine, the most potent H_2_O_2_ inductors under our experimental conditions. As shown in **Figure 4**, carbonyls were clearly detectable in the HL-1 cells incubated with both agents after 1 h, and remained detectable for up to 24 h.

In order to verify whether angiotensin II or phenylephrine induced the carbonylation of M-CK protein, protein extracts from control cells, and cells treated with angiotensin II or phenylephrine, were immunoprecipitated with a specific mouse antibody against M-CK cross-linked to Bio-Adembeads Protein G to avoid the recognition of the heavy and light chain of immunoglobulins in the primary antibody by the second antibody used for Western blotting. After elution, the protein carbonyl groups were derivatised with DNPH before being analysed by Western blotting using an anti-protein-DNP antibody and a rabbit anti M-CK antibody.

**Figure 5:**
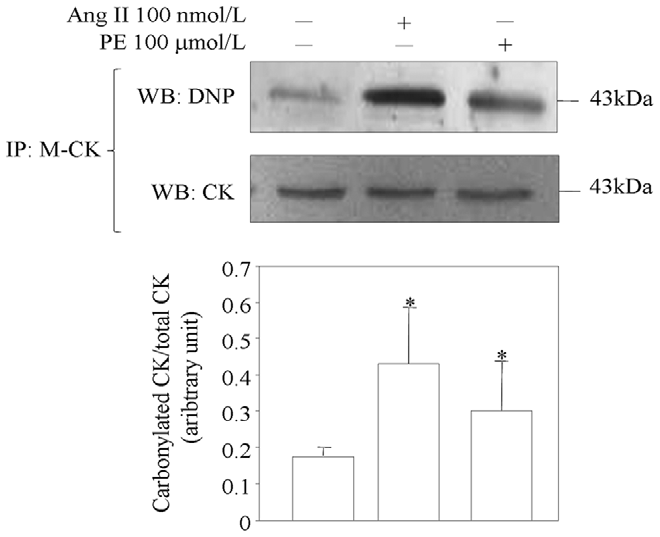
Detection of protein carbonyls after immunoprecipitation of M-CK from cell lysate. HL-1 cells were treated with angiotensin II (AngII) or phenylephrine (PE) for 24 h, and the immunoprecipitates were immunoblotted with anti-DNP and anti-CK. Representative of three independent experiments.

In comparison with the controls, M-CK protein carbonylation was significantly increased by 2.5±0.9 fold in the angiotensin II-treated cells (p<0.05) and by 1.8±0.7 fold in the phenylephrine-treated cells (p<0.05) (**Fig. 5**).

**Figure 6:**
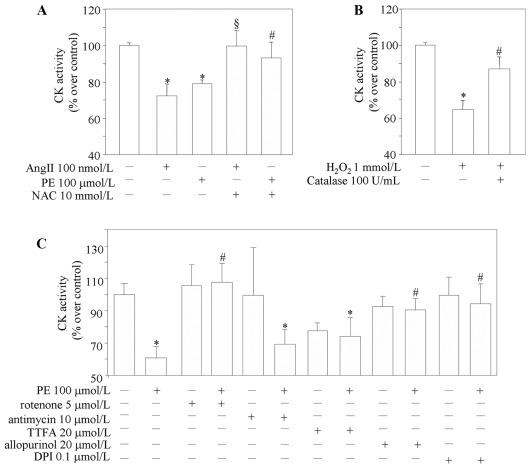
CK activity in cardiomyocytes. (**A**) CK activity in cell lysate measured after pretreatment with NAC (N-acetyl-l-cysteine) for 1 h, and then with angiotensin II (AngII) or phenylephrine (PE) for 4 h in the absence or presence of NAC. (**B**) Oxidative modification of CK activity *in vitro*. Purified human M-CK (30 units/mL) was incubated for 1 h at 25°C with H_2_O_2_ (1 mmol/L) in 25 mmol/L Tris-HCl (pH 7.4) in the absence or presence of 100 units/mL of catalase before measurement of CK activity. *p<0.05 *vs* CK alone. (**C**) CK activity in cells lysate measured after pretreatment with different ROS inhibitors for 1 h, and then with phenylephrine (PE) for 4 h. *p<0.05 *vs c*ontrol; ^#^p<0.05 *vs* PE-treated cells, ^§^p<0.05 *vs* AngII-treated cells (n = 5).

As ROS can oxidatively modify proteins altering their biological activity, [14], [18] we investigated the relationship between the oxidative modification and CK catalytic activity, which was assessed in HL-1 cells treated with angiotensin II or phenylephrine in the absence or presence of antioxidants. The two agents inhibited enzymatic activity, whereas pre-treatment with N-acetyl-l-cysteine (NAC) blocked the decrease induced by both compounds (**Fig. 6A**). We next confirmed that the oxidative modification directly altered the activity of purified CK in *in vitro* conditions: H_2_O_2_ reduced the activity of purified human M-CK, in the absence but not in the presence of catalase (**Fig. 6B**). Taken together, these data suggest that the oxidative modification may be responsible for the loss of M-CK activity in cardiomyocytes exposed to stimuli relevant to HF. The xanthine oxidase inhibitor allopurinol, the NADPH-oxidase inhibitor diphenyliodonium (DPI), and the mitochondrial complex I inhibitor rotenone completely prevented the inhibition of CK activity induced by phenylephrine (**Fig. 6C**). By contrast the complex III inhibitor antimycin and the complex II inhibitor 4,4,4,-trifluoro-1[2-thienyl]-1,3-butanediol did not recover the loss of CK activity (**Fig. 6C**).

## Discussion

Our findings show that oxidative stress-induced protein modifications are increased in the myocardium of HF patients, and the proteomic approach made it possible to ascertain that two proteins mainly underwent carbonylation, ACTC and, to a greater extent M-CK. Moreover, we found that CK activity was significantly impaired in the myocardium of HF patients. The exposure of *in vitro* cultured cardiomyocytes to stimuli relevant to the physiopathology of HF led to ROS generation, the source of which were mainly by xanthine oxidase, NADPH-oxidase, and mitochondrial complex I. This phenomenon induced cell protein carbonylation and the oxidation of CK, which subsequently lost its enzymatic activity. Given the consequences of oxidative modification on protein function, we can hypothesise that the oxidised biomolecules are not only surrogate markers of oxidative stress, but may also play a role in the development and/or progression of HF.

We enrolled HF patients with ischemic or idiopathic dilated cardiomyopathy. The observation that there was no difference between the two HF groups in terms of cardiac protein carbonyl content suggests that the enhanced oxidative stress occurs regardless of the etiology of HF. However, as our study population was small, we may have missed a minor difference. Moreover, we studied patients with end-stage chronic HF, and so we cannot exclude the possibility that protein carbonyl content may be different in the early stages of HF and be related to etiology.

The analysis of protein carbonyls has some advantages over analysing lipid peroxidation products because the formation of protein-bound carbonyl groups seems to be frequent, and oxidised proteins are formed relatively early and are relatively stable. It is known that cells degrade oxidised proteins over hours if not days, whereas lipid peroxidation products are detoxified within minutes. Interestingly, protein carbonyl groups form early and are present for longer than other markers of oxidative stress [23], and the chemical stability of protein carbonyls makes them suitable targets for laboratory measurements [24]. Many different physiological and environmental processes can promote the generation of ROS, including a number of free radicals, various non-radical oxygen derivatives, and highly reactive lipid- or carbohydrate-derived carbonyl compounds. Finally, carbonyl groups are only produced in the presence of severe oxidative stress, and are considered a sign of both oxidative stress itself and protein dysfunction [14]. However, carbonyls are not an index of all oxidative protein modifications, such as the conversion of tyrosine residues to 3-chlorotyrosine, 3-nitrotyrosine, dityrosine or methionine oxidation [14], and so the possibility that other types of modification may occur in patients with HF cannot be excluded. Nitrotyrosine has so far been found to be mainly present in a single protein corresponding to SERCA2a in the failing heart [25].

Oxidative damage often leads to the loss of specific protein function [16]. The inter-relationships of protein oxidation, protein dysfunction and diseases are still unclear, but it is known that oxidative changes in enzymes and structural proteins play a significant role in the pathophysiology of diseases such as Alzheimer’s disease [26] and atherosclerosis [27].

One of the main findings of the present study is that M-CK undergoes carbonylation in the myocardium of HF patients. CK is a major enzyme involved in energy maintenance and transfer in muscle and brain cells, and catalyses the reversible transfer of a phosphate moiety between ATP and creatine. It consists of hetero- and homodimers of two subunits, B and M, and CK isoenzymes are specifically distributed in cardiac cells with the predominant myofibrillar-bound M-isoenzyme, whereas the B-forms contribute little to overall CK activity [28]. MM-CK has been found in myofibrils and described as a structural protein of the M band participating in connections between myosin filaments inside muscle fibers [29]. This bound MM-CK is functionally coupled to the myosin ATPase, and can provide enough energy to sustain maximal force and the normal kinetics of contraction [30].

It has been recently shown that patients with HF show a generalized alteration in energy metabolism, particularly the CK system, with a decrease in total enzyme activity and velocity, and an altered isoenzyme pattern that could contribute to the pathogenesis of HF [31]. This was also confirmed by our finding that CK activity is impaired in the myocardium of HF patients. Moreover, in patients with dilated cardiomyopathy, the phosphocreatine (PCr)/ATP ratio, which is governed by CK activity, may predict both total and cardiovascular mortality [32]. However, the precise cellular mechanisms by which altered CK impairs energy fluxes and contractility are not clearly understood. In addition to alterations in protein abundance, CK activity may also be affected by post-translational modifications, including oxidation. A number of studies have shown that the *in vitro* exposure of CK to ROS or derivatives of nitric oxide, such as peroxynitrite, inhibits CK activity as a result of changes in critical residues within the enzyme [33]. Our hypothesis that oxidative modifications may be behind the reduction in CK activity in the failing heart is supported by the findings presented in this study that the ratio of carbonylated CK to total CK is higher in the myocardium of HF patients; that pro-oxidant stimuli induce CK oxidation in *in vitro* cultured cardiomyocyte, and concomitantly decrease its enzymatic activity; and that the antioxidant NAC and inhibitors of ROS prevent the loss of CK activity.

The enzyme inactivation induced by protein carbonylation may be due to various mechanisms. The active site of CK isoenzymes contains an essential cysteine residue and tyrosine residues that could be targets for the oxidative modifications [34]. The oxidative inactivation of CK might involve the direct free radical-mediated oxidation of these aminoacid residues at the CK site to which ATP binds, a modification that may be due to the reaction of hydrogen peroxide with small amounts of transition metals that can bind to histidines near the active site [35]. Alternatively, the interactions of essential amino acids with the reactive di- or polyaldehyde compounds generated as a result of oxidative stress in a failing heart may block ATP binding to CK, and lead to the introduction of carbonyl groups into CK protein [36]. Oxidative modifications involving carbonylation and the loss of CK activity have been previously demonstrated in the brains of patients with Alzheimer’s disease [37] and in the limb muscles of patients with chronic obstructive pulmonary disease [38].

As our data indicate that CK is a target of ROS in the failing heart, oxidative changes in CK that impair its enzymatic activity may help to explain the decrease in CK activity and consequent defects in energy metabolism associated with HF.

Considering that the nitroso/redox balance is an emerging regulator of cardiovascular homeostasis [39], we have also assessed the occurrence of S-nitrosylation in the human failing heart.

S-Nitrosylation is a reversible protein modification in which nitric oxide is covalently bound to a thiol group, leading to the formation of *S*-nitrosothiols. The relevance of protein nitrosylation is currently under debate. Indeed, it plays an important role in a wide range of NO-mediated cardiovascular effects, including but not limited to mitochondrial metabolic regulation, intracellular Ca^2+^ handling, protein trafficking, and regulation of cellular defense against apoptosis and oxidative stress. However, depending on the localization of NO/SNO signaling, the level of protein S-nitrosylation, and/or interaction with other signaling pathways, the overall effect of protein S-nityrosylation can be protective or detrimental. Furthermore, it has been suggested that S-nitrosylation can protect reactive thiol groups from more irreversible oxidation [40].

Inactivation of CK activity by S-nitrosylation was observed primarily *in vitro*, whereas Decking *et al*. demonstrated [41] that, in the intact guinea pig hearts, the NO-induced reduction in cardiac energy status was associated with an increase in mitochondrial NADH and is not due to inhibition of CK.

On the other hand, in a relevant HF model, the SHHF rat, the global *S*-nitrosylation was decreased in failing hearts compared with nonfailing hearts, and, specifically, the cardiac RyR2 is hyponitrosylated due to nitroso-redox imbalance. Xanthine oxidase inhibition restored global and RyR2 nitrosylation and reversed the diastolic SR Ca^2+^ leak, improving Ca^2+^ handling and contractility [42].

Our results indicate that the pattern of S-nitrosylated proteins was similar in the non failing and failing heart confirming the results by Canton *et al* [43], and, in particular, CK did not display a different nitrosylation status in the failing heart.

Thus, we can conclude that in the human failing heart CK mainly undergoes carbonylation.

However, it is difficult to assess if CK carbonylation mediates functional and metabolic alteration that lead to HF or is an epiphenomenon that reflects cardiomyocytes injury.

Although to a lesser extent than CK, oxidation also affects ACTC, the main component of the thin filament of the sarcomere in cardiac myocytes.

The number and distribution of cytoskeleton elements are altered in a sub-population of cardiomyocytes in hypertrophied [44] and failing myocardium [45], which suggest that myofibrillar and thin filament changes may contribute to the contractile deficiencies associated with human myocardial failure. However, isoform shifts in thin and thick filament proteins are probably not sufficient by themselves to be considered major factors in myofilament remodelling. It is likely that post-translational changes in thin filament proteins play a functional role in some forms of cardiomyopathy because increased levels of circulating troponin I suggesting the ongoing proteolitically degradation of troponin have been observed in patients with severe HF and no clinical evidence of ischemia [46]. Another important potential contributor is contractile protein phosphorylation, which can be altered as a result of changes in kinase and/or phosphatase activity, and our findings now add protein carbonylation to the list of post-translational modifications targeting actin in the failing myocardium.

Given their abundance in mammalian cells, it is likely that actin filaments are common targets for a variety of ROS and reactive carbonyl species [47]. A significant increase in the carbonyl content of β-actin has been found in human brain regions severely affected by Alzheimer’s disease [48], in macrophages exposed to hyperoxia [49], and in the skeletal muscles of a diabetes model in rat [50]. Furthermore, actin carbonylation leading to the disruption of the actin cytoskeleton and the loss of monolayer barrier function has been found in human intestinal cells following exposure to an oxidant insult [51], as well as in the colon mucosa of patients with Crohn’s disease [52].

Furthermore, oxidised sarcomeric and non-sarcomeric actin isoforms have been found in association with significantly depressed post-ischemic contractile function in isolated rat hearts [53] and, more recently, in human hearts, with an inverse correlation to LVEF [43]. Recent data have shown that actin Cys374 may partially act as a scavenger of α,β-unsaturated aldehydes and other electrophilic lipids containing an unsaturated carbonyl group because of its accessible surface and the substantial thiol acidity due to the particular microenvironment surrounding it [54]. Its reactivity against reactive electrophilic carbonyls and its relatively high concentration in comparison with other potential nucleophilic protein targets may explain why oxidised actin was also found in control hearts [55]. Our and others’ previous observations [43], [47] raise the possibility that actin carbonylation may be a mechanism by which oxidative stress controls the organisation of the actin cytoskeleton and regulates the actin dynamics leading to pathological functional impairments.

In the present study functional protein carbonyl groups were analyzed with a highly sensitive assay that involved derivatization with 2,4-dinitrophenylhydrazine (DNPH) to yield a stable 2,4-dinitrophenylhydrazone-protein (DNP-protein) [14] together with two-dimensional electrophoresis and immunoblotting which has been recently reviewed and it is still considered a good approach for the study of oxidized proteins in proteomics [56]. However, our study did not identify the exact amino acid sites of carbonyl modification, which could be necessary for a deeper understanding of the oxidative mechanisms leading to the protein modification. Despite the introduction of many different methods [57], none of them reliably identifies all of the carbonylated amino acid sites, which are not very abundant on endogenously modified proteins; this highlights the need to develop even more sensitive methods.

Nevertheless, our data indicate that two cardiac proteins are targets of ROS in the myocardium of HF patients: ACTC and principally M-CK, which loses its enzymatic activity and thus provides a possible explanation of the defective energy metabolism observed in the failing heart. Our previous findings show that also the plasma proteins α-1-antitrypsin and fibrinogen are oxidized in HF patients, and, that their oxidation leads to protein dysfunction; the first loses its protective inhibitory activity against elastase, and the second becomes cytotoxic, two events that may contribute to the endothelial damage associated with heart failure [18]. Overall, these data suggest that altered oxidation pathways observed in HF can lead to specific post-translational modifications of relevant proteins and thus contribute to the disease.

Finally, proteomic studies allow a much wider view of oxidative stress responses than conventional biochemical methods and, together with dedicated biochemical tools and mass spectrometry-based quantitative techniques, will make it possible to study changes in the expression and oxidative modifications of unpredictable proteins.

### Limits of the Study

Few study limitations should be acknowledged. First, our study involved a small number of cases and studies of larger population are certainly needed. Secondly, as we used myocardium obtained from patients undergoing cardiac transplantation, our results apply to subjects with real end-stage heart failure. No data are available concerning patients with less severe heart failure, and so the progression of myocardial protein oxidation during the natural evolution of heart failure is unknown.

Moreover although the protein oxidation in the myocardium of patients with end-stage heart failure seems to be qualitatively and quantitatively important, it is not known whether it is deleterious or not, nor whether it can be modulated by drugs: two aspects that need further investigation in larger patient series. It is well known that although extensive studies have linked free-radical injury to HF, there is currently no antioxidant-based therapy that is being used in patients with this disease. We agree with the Authors of a recent review [58] who assess that to develop effective and safe therapeutic strategies that target oxidative stress, we must first understand the ROS-generating molecular pathways that may be involved in the pathogenesis of HF. It is important to emphasize that in the heart there are complex and dynamic molecular pathways involved in ROS formation. Therefore the inability to elucidate the beneficial effects of antioxidant strategies in humans may be attributed to the fact that only specific ROS-generating pathways may be modifiable or worth modulating. Furthermore, one of the major limitations in administrating antioxidants to prevent ROS generation in myocardial tissue is the difficulty to achieving significant local concentrations of the antioxidants. If, for example, mitochondria are involved in the ROS generation, antioxidants specifically targeted to this local source are needed. Our study shows that xanthine oxidase, NADPH-oxidase, and mitochondrial complex I prevented the loss of CK activity induced by phenylephrine. Therefore, an *in vivo* antioxidant treatment should take into consideration these specific pathways. Indeed, pharmacological strategies are currently examining the role of xanthine oxidase inhibitors, such as allopurinol and oxypurinol, in targeting oxidative stress in HF [58]. Studies in animal models of HF have demonstrated that allopurinol reverses left ventricular remodeling, improves LVEF, and reduces endothelial dysfunction in HF. However, clinical studies have yielded inconsistent results probably because it is important to target a specific population of patients with HF; for instance, patients with moderate-to severe HF that had elevated serum uric acid levels are most likely to benefit from allopurinol treatment, the cornerstone of the clinical management of gout and conditions associated with hyperuricemia for several decades [59].

Finally, like most proteomic studies of human samples, our study largely provides a descriptive list of differentially expressed proteins and, although they have the potential value of identifying biomarkers of human disease for clinical use, such studies have not yet provided the substantial mechanistic insights [60] needed to improve our understanding of the physiopathology of the disease. Only when the findings of discovery proteomic studies are used to shed light on the mechanisms of heart disease will they begin to show their full potential, and may allow the development of new therapeutic strategies for cardiac diseases.

In conclusion, our findings show that protein oxidation is increased in the myocardium during HF, but it is still not clear if oxidative stress plays a causal role in heart failure, is an epiphenomenon unrelated to its pathogenesis, or even a consequence of the disease.

## Supporting Information

Figure S1 Detection of S-nitrosylated CK in myocardium from HF patients and controls. (**A**). Nitrosylated proteins have been biotinylated and subjected to immunoprecipitation of creatine kinase (CK). Immunoblots were performed with anti-CK, to confirm the immunoprecipitation, and with an anti-biotin antibody, to identify S-nitrosylated CK. (**B**). The amount of S-nitrosylated CK was given by the ratio between densitometric values of the S-nitrosylated band and those of the CK band. Values are mean±SD. (**C**). Representative 1-DE image of total nitrosylated proteins in myocardium of controls and HF patients. Nitrosylated proteins have been biotinylated and equal amount of proteins have been subjected to immunoblotting with a biotin detection reagent.
(PPTX)

Table S1 LC-MS/MS identification of proteins showing differences in protein carbonylation.
(DOCX)

Methods S1 Supporting information for Methods section.
(DOCX)
