# Supplementary figures and images for: Redox Proteomics Identification of Oxidatively Modified Myocardial Proteins in Human Heart Failure: Implications for Protein Function
Source: PLoS One. 2012 May 14;7(5):e35841. doi: 10.1371/journal.pone.0035841 (PMC3351458; doi:10.1371/journal.pone.0035841)

## Slide 1
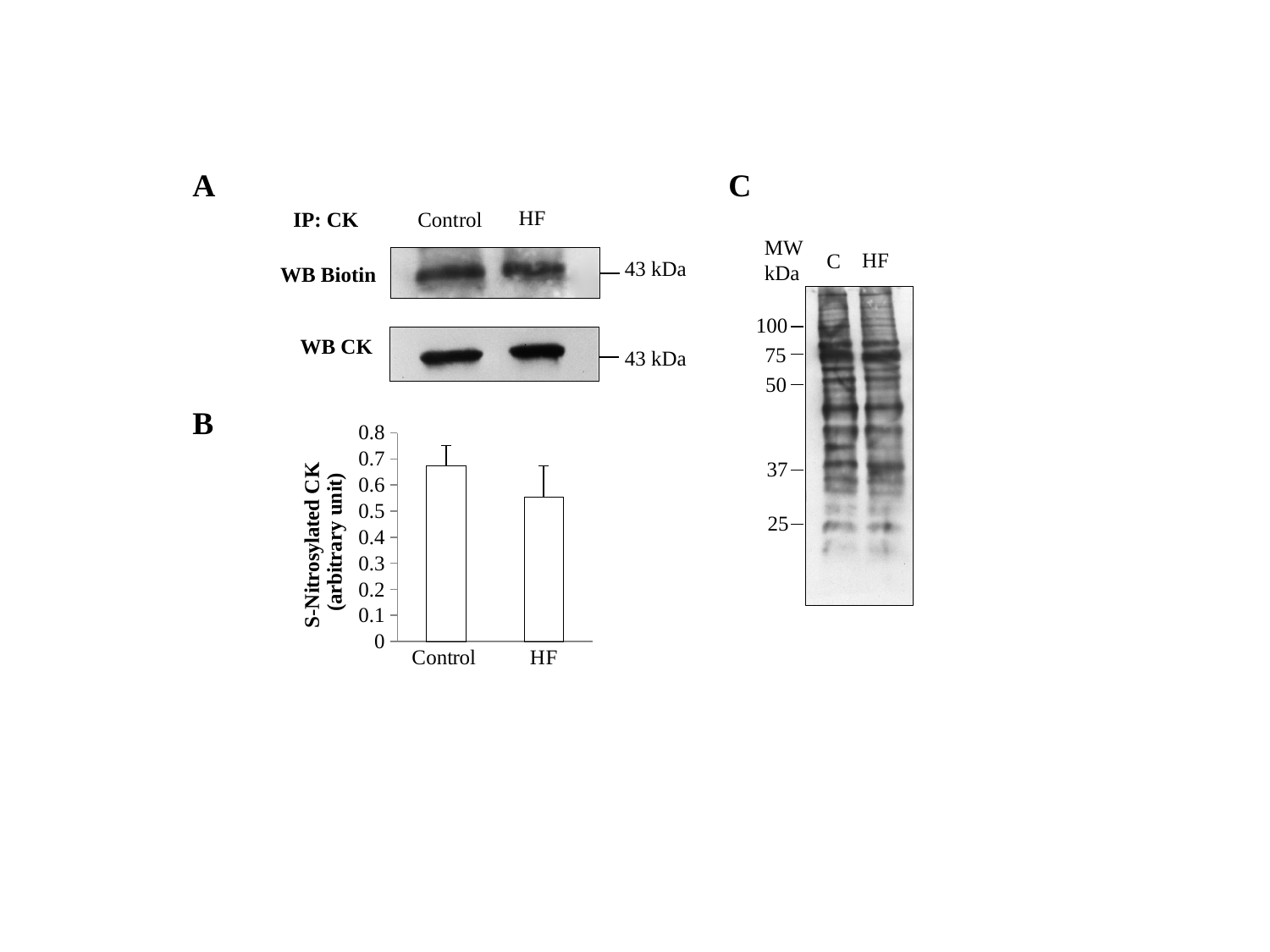

A
C
HF
Control
IP: CK
43 kDa
WB Biotin
WB CK
43 kDa
### Chart
| Category | |
|---|---|
| Control | 0.6718750000000002 |
| HF | 0.5533707865168539 |MW kDa
HF
C
100
75
50
B
37
25

Supplement: Figure S1 — Detection of S-nitrosylated CK in myocardium from HF patients and controls. (A). Nitrosylated proteins have been biotinylated and subjected to immunoprecipitation of creatine kinase (CK). Immunoblots were performed with anti-CK, to confirm the immunoprecipitation, and with an anti-biotin antibody, to identify S-nitrosylated CK. (B). The amount of S-nitrosylated CK was given by the ratio between densitometric values of the S-nitrosylated band and those of the CK band. Values are mean±SD. (C). Representative 1-DE image of total nitrosylated proteins in myocardium of controls and HF patients. Nitrosylated proteins have been biotinylated and equal amount of proteins have been subjected to immunoblotting with a biotin detection reagent. (PPTX) [file pone.0035841.s001.pptx]
