# Supplementary material for: Redox Proteomics Identification of Oxidatively Modified Myocardial Proteins in Human Heart Failure: Implications for Protein Function
Source: PLoS One. 2012 May 14;7(5):e35841. doi: 10.1371/journal.pone.0035841 (PMC3351458; doi:10.1371/journal.pone.0035841)
Supplement: Table S1 — LC-MS/MS identification of proteins showing differences in protein carbonylation. (DOCX) [file pone.0035841.s002.docx]

**TTable S1:** LC-MS/MS identification of proteins showing differences in protein carbonylation.

| No. | Protein name / Accession code^a^ | Oxidation index (fold increase)^b^ | MW  Kda /pI | Sequence coverage  % / Score^c^ | Sequences | Peptide score |
| --- | --- | --- | --- | --- | --- | --- |
| 1a | α-cardiac actin / ACTC_HUMAN | 2.2±0.4 | 48.7/5.23 | 9/51 | (K)AGFAGDDAPR(A)  (K)SYELPDGQVITIGNER(F)  (K)IIAPPER(K)  (K)IIAPPERK(Y)^d^ | 13  9  38  6 |
| 1b | α-cardiac actin / ACTC_HUMAN | 2.5±0.3 | 48.7/5.23 | 11/81 | (R)GYSFVTTAER(E)  (K)SYELPDGQVITIGNER(F)  (K)EITALAPSTMK(I)  (K)IIAPPER(K) | 14  53  28  12 |
| 1c | α-cardiac actin / ACTC_HUMAN | 1.7±0.5 | 48.7/5.23 | 39/520 | (K)AGFAGDDAPR(A)  (K)DSYVGDEAQSK(R)  (K)DSYVGDEAQSKR(G)^d^  (R)VAPEEHPTLLTEAPLNPK(A)  (R)DLTDYLMK(I)  (R)GYSFVTTAER(E)  (K)LCYVALDFENEMATAASSSSLEK(S)  (K)SYELPDGQVITIGNER(F)  (K)SYELPDGQVITIGNER(F)^e^  (K)DLYANNVLSGGTTMYPGIADR(M)  (K)EITALAPSTMK(I)  (K)EITALAPSTMK(I)^e^  (K)IIAPPER(K)  (K)QEYDEAGPSIVHR(K) | 76  49  51  49  25  49  4  60  103  112  23  45  39  96 |
| 2a | Creatine kinase M type KCRM_HUMAN | NC | 43.1/6.77 | 43/586 | (K)LNYKPEEEYPDLSK(H)  (K)VLTLELYK(K)  (K)VLTLELYKK(L)^d^  (K)ELFDPIISDR(H)  (R)HGGYKPTDK(H)  (K)TDLNHENLK(G)  (K)GYTLPPHCSR(G)  (K)GYTLPPHCSR(G)^e^  (K)LSVEALNSLTGEFK(G)  (K)LSVEALNSLTGEFK(G)^e^  (R)DWPDAR(G)  (K)SFLVWVNEEDHLR(V)  (R)RFCVGLQK(I)  (R)FCVGLQK(I )  (K)IEEIFK(K)  (K)FEEILTR(L)  (K)RGTGGVDTAAVGSVFDVSNADR(L)^d^  (K)RGTGGVDTAAVGSVFDVSNADR(L)^d^  (R)GTGGVDTAAVGSVFDVSNADR(L)  (R)GTGGVDTAAVGSVFDVSNADR(L)^e^  (R)LGSSEVEQVQLVVDGVK(L)  (R)LGSSEVEQVQLVVDGVK(L)^e^  (K)GQSIDDMIPAQ(K)  (K)GQSIDDMIPAQ(K)^e^ | 21  9  23  46  8  41  30  2  38  116  14  76  16  10  7  41  7  72  62  128  56  124  12  11 |
| 2b | Creatine kinase M type KCRM_HUMAN | NC | 43.1/6.77 | 68/540 | (K)LMVEME(K)  (K)VLTLELY(K)  (K)GGNMKEVF(R)  (K)TDLNHENL(K)  (K)VLTLELYK(K)  (K)HPKFEEILT(R)  (K)GQSIDDMIPAQ(K)  (K)GGDDLDPNYVLSS(R) (K)LSVEALNSLTGEF(K)  (K)SFLVWVNEEDHL(R)  (K)LSVEALNSLTGEFKG(K) (K)LSVEALNSLTGEFKG(K)^e^ (K)LNYKPEEEYPDLS(K) (K)LNYKPEEEYPDLS(K)^e^ (R)LGSSEVEQVQLVVDGV(K) (R)GTGGVDTAAVGSVFDVSNAD(R) (K)TDLNHENLKGGDDLDPNYVLSS(R) (R)GIWHNDNKSFLVWVNEEDHL(R)  (K)SMTEKEQQQLIDDHFLFDKPVSPLLLASGMA(R)  (K)SMTEKEQQQLIDDHFLFDKPVSPLLLASGMA(R)^e^ | 26  49  52  31  42  71  62  65  52  39  32  96  55  23  46  38  34  37  75  42 |
| 2c | Creatine kinase M type KCRM_HUMAN | NC | 43.1/6.77 | 40/564 | (K)LNYKPEEEYPDLSK(H)  (K)ELFDPIISDR(H)  (R)HGGYKPTDK(H)  (K)GYTLPPHCSR(G)  (K)LSVEALNSLTGEFK(G)  (R)DWPDAR(G)  (K)SFLVWVNEEDHLR(V)  (R)RFCVGLQK(I)^d^  (R)FCVGLQK(I )  (K)IEEIFK(K)  (K)FEEILTR(L)  (R)GTGGVDTAAVGSVFDVSNADR(L)  (R)LGSSEVEQVQLVVDGVK(L)  (K)GQSIDDMIPAQ(K) | 46  18  75  26  24  18  36  89  51  28  27  35  31  26 |

^a^ Swiss-Prot accession code

^b^ ratio between spot immunointensity divided by intensity of protein staining with Colloidal Blue (fold increase in HF patients vs controls , mean±SD; p<0.05 *vs* control).

^c^ Mascot score

^d^ peptide with missed cleavage

^e^ peptide with oxidized methionine

NC = uncalculable because of the undetectable level of carbonylated M-CK in the controls.
