# Supplementary material for: Redox Proteomics Identification of Oxidatively Modified Myocardial Proteins in Human Heart Failure: Implications for Protein Function
Source: PLoS One. 2012 May 14;7(5):e35841. doi: 10.1371/journal.pone.0035841 (PMC3351458; doi:10.1371/journal.pone.0035841)
Supplement: Methods S1 — Supporting information for Methods section. (DOCX) [file pone.0035841.s003.docx]

**SUPPLEMENTARY MATERIALS AND METHODS**

**Sample preparation and 2-dimensional electrophoresis for the detection of protein carbonyls**

The cardiac proteins were diluted in a buffer yielding final concentrations of 8 mol/L urea, 2 mol/L thiourea, 4% w/v CHAPS, 2% v/v carrier ampholytes, pH 3-10, 20 mmol/L Tris, 55 mmol/L DTT, and bromophenol blue. Two-dimensional electrophoresis (2-DE) was carried out in accordance with the manufacturer's protocol (Protean IEF cell, Biorad, Milan, Italy), with 70 mm IPG ready strips, pH 3-10 non-linear gradient (Biorad, Milan, Italy), being actively rehydrated at 50 V for 24 hours. The samples were loaded at the anode and focused for a total of 20 KVh. Following isoelectrofocusing, the oxidised proteins were derivatised using the previously described in-strip DNPH derivatisation method [1]. Briefly, the IPG strips were incubated in 2 N HCl with 10 mmol/L DNPH, washed with 2 mol/L Tris/30% v/v glycerol, and equilibrated first with a solution containing 50 mmol/L Tris-HCl, 6 mol/L urea, 30% v/v glycerol, 2% w/v SDS and 2% w/v dithiothreitol, and then with the same buffer containing 4.5% w/v iodoacetamide instead of dithiothreitol. The second dimension was run on 12% polyacrylamide gels. All of the analyses were made in duplicate: one gel was stained with Coomassie Colloidal Blue G250 for total protein analysis, and the other was electroblotted to a polyvinylidene fluoride (PVDF) membrane for DNP immunostaining to evaluate the carbonylated proteins. The procedure was performed in triplicate for each sample in order to evaluate gel reproducibility and improve the reliability of the estimates of qualitative and quantitative changes in protein expression.

To analyse total proteins, the gels were fixed with a fixing solution containing 40% v/v methanol and 10% v/v acetic acid, stained overnight with a solution containing 0.12% w/v Coomassie Blue G250, 8% w/v (NH_4_)_2_SO_4_, 1.6% v/v phosphoric acid, and 20% v/v methanol, and then destained with a solution containing 25% v/v methanol.

For immunodetection, the membranes were stained in 0.2% w/v Ponceau S in 3% w/v trichloroacetic acid, and the spot position was marked in order to facilitate computer-assisted matching on the Coomassie Blue-stained gel. Immunodetection was performed as previously described [1] using biotinylated anti-DNP antibody (Invitrogen, Milan, Italy). The immunoreactive spots were detected using peroxidase-conjugated avidin horseradish (Biorad, Milan, Italy) and a chemiluminescence detection system (GE Healthcare, Milan, Italy).

All of the images were scanned using a GS-800 densitometer (Biorad, Milan, Italy) before being analysed by means of Progenesis SameSpot software (Nonlinear Dynamics, Newcastle upon Tyne, UK).

Progenesis SameSpot software v 2.1 was used for gel alignment, spot detection, spot quantification, and normalisation for total spot volume in each gel, and the data were statistically analysed using the incorporated statistical package. Oxidation index was then calculated as the ratio between spot immunointensity divided by intensity of Colloidal Blue protein staining. The cut-off level for a differentially expressed protein was defined as at least a 1.5 fold increase or decrease in spot intensity.

Statistically significant between-group differences for each protein were computed using the non-parametric Wilcoxon Mann-Whitney test, and a p value of <0.05 was considered statistically significant. Proteins with altered immunoreactivity were identified by mass spectrometry (MS) after excision of the matching spot on the superimposed gel stained with Coomassie Blue.

**Mass spectrometry analysis**

The selected proteins were in-gel reduced, alkylated, digested with trypsin as previously described [2] and the samples were analysed by liquid chromatography-mass spectrometry (LC-MS/MS)-, with the spectra being recorded by a hybrid quadrupole orthogonal acceleration time-of-flight Q-Tof mass spectrometer with an electrospray ionization (ESI) source, Synapt-MS (Waters corporation, Manchester, UK), connected to a Nano-Acquity UPLC system. The samples were dissolved in 0.1% v/v formic acid, and aliquots were injected onto a 180µm x 20 mm Symmetry C18 trap column (Waters corporation, Manchester, UK) for preconcentration and desalting. The samples were subsequently directed from the precolumn onto a 75 μm inner diameter × 250 mm C_18_ nanoAcquity™ UPLC™ column (1.7 μm particle size; Waters corporation, Manchester, UK) and the elution was performed at a flow rate of 300 nL/min by increasing the organic solvent concentration from 1 to 40% B in 30 min, using 0.1% formic acid in water as reversed phase solvent A and 0.1% formic acid in acetonitrile as reversed phase solvent B. The time-of-flight analyzer of the mass spectrometer was externally calibrated with NaI from *m*/*z* 50 to 1990, with the data post-acquisition lock mass corrected using the mono-isotopic mass of the doubly charged precursor of [Glu^1^]-Fibrinopeptide B. The latter was delivered at 250 fmol/μL to the mass spectrometer via a NanoLockSpray interface at a flow rate of 200 nL/min and the reference sprayer was sampled every 30 s. The capillary voltage was set to 3000 V. A survey scan over the m/z range of 350–1990 was used to identify protonated peptides with charge states of 2, 3 or 4, which were automatically selected for data-dependent MS/MS analysis (Mass links v4.1 SCN639, Waters). All raw MS data were processed with PLGS software (version 2.3.23, Waters) and the proteins were identified by correlating the uninterpreted spectra with entries in Swiss-Prot/TrEMBL. A UniProtKB/Swiss-Prot database (release 57; 03/2009; number of human sequence entries, 20334) was used for database searches of each run. Methionine oxidation was considered a variable modification, one missed cleavage per peptide was allowed, and the mass tolerance window was set to 20 ppm for peptides and 0.05 Da for fragments.

In parallel, the spectra were also searched against the National Center for Biotechnology Information non-redundant (NCBI nr) database using Mascot (Matrix Science). Valid identification required two or more peptides independently matching the same protein sequence, a significant peptide score, and the manual confirmation of agreement between the spectra and peptide sequence. In addition, Mascot searches of all spectra were performed against a randomised version of the NCBI database using the same parameters as in the main search.

**References**

1. Banfi C, Brioschi M, Barcella S, Veglia F, Biglioli P, et al. (2008) Oxidized proteins in plasma of patients with heart failure: role in endothelial damage. Eur J Heart Fail 10: 244-251.
2. Banfi C, Brioschi M, Wait R, Begum S, Gianazza E, et al. (2005) Proteome of endothelial cell-derived procoagulant microparticles. Proteomics 5: 4443-4455.
